# Team Interdependence as a Substitute for Empowering Leadership Contribution to Team Meaningfulness and Performance

**DOI:** 10.3389/fpsyg.2022.637822

**Published:** 2022-02-11

**Authors:** Alon Lisak, Raveh Harush, Tamar Icekson, Sharon Harel

**Affiliations:** ^1^Department of Management, Guilford Glazer Faculty of Business and Management, Ben-Gurion University of the Negev, Be’er Sheva, Israel; ^2^The Graduate School of Business Administration, Bar-Ilan University, Ramat Gan, Israel; ^3^School of Behavioral Sciences, Peres Academic Center, Rehovot, Israel; ^4^Department of Education, Ben-Gurion University of the Negev, Be’er Sheva, Israel

**Keywords:** empowering leadership, team meaningfulness, task interdependence, team performance, substitute for leadership

## Abstract

This study uses a relational work design perspective to explore substitutes for leadership behaviors that promote team meaningfulness and performance. We propose that team task interdependence, a structural feature facilitating interaction among team members, can be a substitute for the contributions of empowering leadership. Data were collected from 47 R&D and technology implementation teams across three organizations in a cross-sectional field study. The results revealed that high task interdependence attenuated the contributions of empowering leadership concerning team meaningfulness and, indirectly, to team performance. These findings highlight that the importance of leaders as generators of team meaningfulness is contingent on team relational work design.

## Introduction

The quest for meaningfulness is central to employees who strive to make their work purposeful and to organizations that aim to improve their outcomes (Martela and Pessi, 2018). Ample evidence from organizational psychology research indicates that employees’ sense of meaningfulness with regard to work can contribute positively to organizational outcomes, such as job satisfaction, engagement, commitment, citizenship behaviors, and organizational performance (e.g., Rosso et al., 2010; Schnell et al., 2013; Michaelson et al., 2014). As a team phenomenon, meaningfulness refers to the “level at which team members perceive their teams’ tasks as important, valuable, and worthwhile for their organizations” (Kirkman and Rosen, 1999 p. 59). The ability to create and maintain a high level of team meaningfulness is an asset to teams and organizations, as it can facilitate team performance (Kirkman et al., 2004a; Lee et al., 2018a). Thus, cultivating team meaningfulness is a central aim of most organizations, especially under the prevalent practice of using team-based work structures (e.g., Ilgen et al., 2005; Mathieu et al., 2017).

**FIGURE 1:**
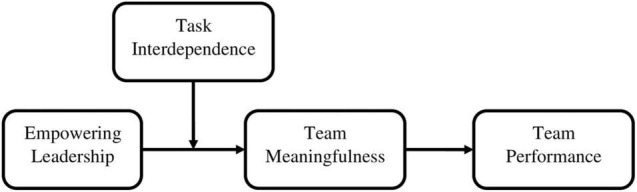
Research model.

Nevertheless, research on cultivating meaningfulness at the team level is scarce. Most such research deals with ways in which leaders can foster meaningfulness as part of team psychological empowerment (Kirkman and Rosen, 1999; Kirkman et al., 2004a,b; Chen et al., 2007; Spreitzer, 2008). This leadership research builds on the Job Characteristics Theory (JCT; Hackman and Oldham, 1980), which describes how leaders initiate and design job characteristics such as skill variety, task identity, and task significance to enhance work meaningfulness. Team meaningfulness, however, is defined as a collective team construct that involves team members’ collective perceptions of their tasks (Kirkman and Rosen, 1999). Although team meaningfulness is defined by the collective perception of members, team-related literature has not yet turned its attention to the possible role of relational work design, which allows the team members to interact with others and affects their attitudes to work, in creating team meaningfulness (Grant, 2007; Grant and Parker, 2009). In this study, we address this research gap by taking the *relational work design* perspective, “which focuses on how work structures can provide more or fewer opportunities for employees to interact with others, which in turn affect their motivation, attitudes, and job performance” (Parker, 2014, 668), to suggest that interaction levels of team members contribute to their team meaningfulness. Specifically, we focus on *task interdependence*, that is, the extent to which team members depend on one another to carry out work effectively (Van der Vegt and Janssen, 2003), as a structural feature that enhances interaction (Courtright et al., 2015). We integrate this idea into a research model that relies on the framework of substitute for leadership theory (Kerr and Jermier, 1978; Howell et al., 1986), which delineates how task-related factors, along with other organizational factors, can act as substitutes for the effect of leaders’ behaviors on individual and team outcomes. In this research model, we explore how task interdependence of team members can substitute the effect of empowering leadership behaviors on team meaningfulness and, consequently, on team performance (see Figure 1). The idea that task interdependence of members can act as a substitute for empowering behavior of leaders is in line with previous ideas from the substitute for leadership theory, suggesting that high interdependence within teams (“closely-knit” teams) enables the direct provision of task-relevant guidance and feedback by the primary members, thus serving as a substitute for formal leadership activities (Kerr and Jermier, 1978; Howell and Dorfman, 1986).

By relying on ideas from the relational work design perspective and substitute for leadership, this study contributes to the leadership literature. We address recent calls to explore factors that can replace empowering leadership and extend the knowledge on them (Sharma and Kirkman, 2015; Lee et al., 2018a; Cheong et al., 2019) by demonstrating that team task interdependence is a structural feature that can substitute empowering contribution of leaders to team meaningfulness.

Moreover, we contribute to the literature on work meaningfulness by focusing on team task interdependence as a feature that can promote task-related interactions between members as a substitute for empowering leadership behaviors. As a result, the dependency on empowering leadership behavior as a source of team meaningfulness is reduced, while alternative routes of fostering team meaningfulness are identified. Finally, we contribute to the emerging literature on relational work design, which initially focused on interactions with service beneficiaries outside the organization (e.g., Grant et al., 2007; Grant, 2008b) by extending its scope to include interactions with employees within organizational teams.

## Literature Review and Hypothesis Development

### Individual and Team Meaningfulness at Work

Meaningfulness is a fundamental human need (Baumeister, 1991). Frankl (1992) argued that seeking meaning at work is a primary motive, and Cascio (2003) identified meaningful work as the most crucial feature of any job. Similarly, Seligman (2002) suggested that meaningfulness enables individuals to find purpose, significance, and importance in their work. Studies show that perceptions of work meaningfulness contribute to job satisfaction, commitment, citizenship behaviors, and organizational performance of employees (e.g., Rosso et al., 2010; Michaelson et al., 2014). Conversely, a lack of work meaningfulness can lead to apathy, disengagement, and alienation (Thomas and Velthouse, 1990). Research has traditionally explored work meaningfulness as part of the job design approach, examining how task and relationship design can affect the willingness of employees to invest time and effort in performing their job effectively (Ilgen and Hollenbeck, 1991). This stream of research focuses on work meaning created by individual-level factors, job dimensions, and the fit between the two (Hackman and Oldham, 1980; Kulik et al., 1987).

Team meaningfulness is a collective construct that corresponds to meaningfulness at the individual level, and entails perceiving team tasks as essential and worthwhile (Kirkman and Rosen, 1999; Chen et al., 2007). Team members who experience team meaningfulness possess a strong collective commitment to their mission, work with a sense of purpose, and share a strong belief in the importance of their team’s cause (Kirkman and Rosen, 2000). Moreover, they regard even the most trivial parts of their jobs as being integral to the overall success of the team; therefore, they can effectively “experience ordinary tasks in an extraordinary way” (Kirkman and Rosen, 2000, 50). As with other team-related collective constructs, team meaningfulness emerges from a series of ongoing events and interactions between members (Morgeson and Hofmann, 1999). Members tend to have a direct effect on other members’ experiences of meaningfulness, as they interactively develop and share the meaningfulness of their team tasks (Kirkman and Rosen, 1999). Meaningfulness is related to three core job characteristics: skill variety (i.e., the varied activities, skills, and talents required to execute the work); task identity (i.e., designing the work as a whole, that is, as an identifiable piece from beginning to end); and task significance (i.e., connecting the job to its impact on other people’s lives; Oldham and Hackman, 2010). Each of these job characteristics has been addressed at the team level and discussed as part of teamwork processes. Skill variety is related to cross-training for team jobs of other members, thus providing a greater level of experience and flexibility. Task identity is linked to the involvement of team members in customer provision, or with a complete product or service. Task significance has been connected to the intrinsic motivation of members to work due to its effect on other team members, and because it provides a more complete understanding of team tasks and their importance to the organization based on the information provided by the team network (Kirkman and Rosen, 1999).

### Cultivating Team Meaningfulness and Performance by Empowering Leadership

Most of the current research has viewed leaders as being the initiating force shaping employees’ experiences of meaningfulness (e.g., Shamir et al., 1993; Luthans and Avolio, 2009). The majority of this research focuses on the individual level and explores how leadership behaviors, such as transformational leadership (Piccolo and Colquitt, 2006; Oh and Roh, 2019), ethical leadership (Wang and Xu, 2019), and empowering leadership (e.g., Kim et al., 2018; Lee et al., 2018a; Gao and Jiang, 2019) foster meaningfulness in the jobs of followers. Only limited research has examined the influence of leadership on team meaningfulness (Yang et al., 2019), and it has mostly been examined as part of team empowerment research. This research shows that empowering leaders can enhance team psychological empowerment (including team meaningfulness) through behaviors such as delegation of responsibility, informed and participative decision making, coaching, goal setting, showing concern for and confidence in high team performance, and enhancing autonomy regarding bureaucratic constraints. From all these empowering behaviors, team meaningfulness is most directly related to leadership behaviors such as providing information about the meaning of the team task to team members, and encouraging participation of team members in decision-making processes that enhance their sense of care toward team tasks (Kirkman and Rosen, 1999; Chen et al., 2007; Fong and Snape, 2015).

The ability of leaders to foster team meaningfulness has implications for improving team performance. Work meaningfulness is a critical psychological state for developing internal work motivation, which enhances performance (Hackman and Oldham, 1980). Research shows that when employees perceive their jobs as meaningful, and their responsibilities as impacting others, they are more motivated to perform well (Liden et al., 2000; Wrzesniewski et al., 2003). Similarly, teams with higher levels of empowerment, and specifically team meaningfulness, enhance team performance (e.g., Kirkman et al., 2004a; Chen et al., 2007; Yang et al., 2019). Such team empowerment increases task motivation due to team members’ collective and positive assessments of their organizational tasks (Kirkman et al., 2004a). Team members who share a high sense of team meaningfulness make efforts to understand a problem from diverse points of view, use a wide variety of information sources to search for a solution, generate a significant number of alternatives, improve the quality of their work, and demonstrate high team productivity and performance (Srivastava et al., 2006; Park et al., 2017). Previous studies have not tested the mediating role of team meaningfulness in the relationship between empowering leadership and team performance. However, they demonstrated such a relationship in conjunction with team empowerment (Lee et al., 2018a). Thus, we propose that empowering leadership will foster team meaningfulness, which, in turn, will positively affect team performance.

*Hypothesis 1: There is a positive indirect relationship between empowering leadership and team performance through team meaningfulness.*

### Relational Work Design, Task Interdependence, and Team Meaningfulness

Cultivating experiences of meaningfulness were historically explored as part of the motivational work design approach, commonly represented by the job characteristic model that focused on five core structural characteristics of jobs (task variety, autonomy, feedback, significance, and identity; Hackman and Oldham, 1980). Over time, scholars started to broaden the focus beyond the historically narrower emphasis on the core aspect of the job, to include other aspects of work, and their relevance to team meaningfulness (e.g., Parker et al., 2001; Campion et al., 2005). One of these work design aspects is the social context of work, which refers to interpersonal interactions and relationships that are embedded in and influenced by the jobs, roles, and tasks that employees perform, and play a critical role in shaping experiences and behaviors of employees (Grant and Parker, 2009). This link between interactions and relationships of individuals at work, and their work-related attitudes and outcomes, lies within the emergent viewpoint of relational work design that focuses on how roles are designed to enhance opportunities for employees to positively interact with others (Parker, 2014). Research within the relational work design perspective found that workers who interact with others perceive their work as being impactful, and increase their task significance and performance (Grant et al., 2007; Grant, 2008a,b). Task significance is also one of the core job characteristics that most directly facilitates work meaningfulness (Oldham and Hackman, 2010), and in this line of thought, relational work design has been identified as a path for increasing the meaningfulness of work (Parker, 2014). Scholars suggested that such positive interactions and relationships influencing the meaning of work could be shared with other persons or groups both outside the organization (e.g., service beneficiaries; Grant et al., 2007; Grant, 2008b) and within it (e.g., other workers; Rosso et al., 2010; Parker et al., 2013). One specific form of work that can benefit from relational work design aspects is teamwork. Relational work designs that enhance social interactions between team members are likely to enable the emergence of collective phenomena such as team meaningfulness (Morgeson and Hofmann, 1999; Courtright et al., 2015).

One aspect of relational work design that increases interaction opportunities is work interdependence (Grant and Parker, 2009; Courtright et al., 2015). Following the trends of globalization, technological change, the shift toward a service and knowledge economy, and a greater proportion of teamwork, work has become more interdependent than ever before (Grant and Parker, 2009; Parker et al., 2013). In this study, we focus on the interdependency of teamwork in the form of team task interdependence, that is, the extent to which team members rely on one another to effectively fulfill their work-related demands (Courtright et al., 2015). High task interdependence requires team members to cooperate and work interactively to accomplish their tasks (Campion et al., 1993; Stewart and Barrick, 2000; Van der Vegt and Janssen, 2003). In terms of team processes, task interdependence enhances reciprocal task-focused interactions of members, such as process planning and orchestrating taskwork, to accomplish the team task (Courtright et al., 2015). Grant and Parker (2009) highlighted interdependence as a central factor that shapes work design and its outcomes. They portrayed task interdependence as a critical social characteristic of work that affects relational and emotional mechanisms such as perceived impact, interpersonal cohesion, and affective interpersonal commitment, all of which are linked to outcomes of motivation and performance, attitudes, team coordination, and cognition. Considering the effect of interaction with others on the perceived impact of work, we suggest that as was found when employees interacted with beneficiaries outside the organization (e.g., Grant et al., 2007; Grant, 2008b), task interdependence is likely to contribute to team meaningfulness by providing an understanding of the task significance of the team to beneficiaries within the organization. High interdependence allows team members to see how their tasks are connected, providing a more comprehensive understanding of the team’s task impact and importance to the organization, which is the core of team meaningfulness (Kirkman and Rosen, 1999). Another aspect of task interdependence that contributes to team meaningfulness is task identity. To successfully accomplish an interdependent team task, members enhance their interactions through planning and orchestrating taskwork (Courtright et al., 2015). Such processes connect all aspects of the team task and provide more complete information about the task as a whole. Thus, tasks of team members and their interconnections become identifiable from beginning to end, enhancing team task identity, and the team’s perception of being important to the organization. Finally, high team interdependence and more team member interactions can provide opportunities to enhance meaningfulness by strengthening members’ sense of team identity, and their feelings of belonging to the team (Ashforth and Mael, 1989; Pratt and Ashforth, 2003). Thus, we explore the possibility that high team task interdependence, characterized by intense social interaction between members, works in a similar direction to that of empowering behaviors of leaders. In such a situation, high levels of team task interdependence will likely attenuate the effect of empowering behaviors of leaders have on team meaningfulness.

Exploring task interdependence as an enabler of team meaningfulness is in line with the substitutes for leadership theory (e.g., Kerr and Jermier, 1978; Howell and Dorfman, 1986; Howell et al., 1986; Dionne et al., 2005). Substitutes for leadership are factors that replace leadership behaviors and diminish or attenuate the ability of leaders to influence subordinate criterion variables (Kerr and Jermier, 1978). In such cases, although both leadership behaviors and the substitute act in the same direction concerning the outcome, the interaction between them occurs in the opposite direction, thus reflecting a situation where a potent substitute attenuates the relationship between leadership behavior and the outcome (Howell et al., 1986; Dionne et al., 2005). Limited research has examined substitutes for empowering leadership and their influence on team outcomes, specifically team meaningfulness and performance (Cheong et al., 2019). We claim that task interdependence can serve as a substitute for leadership, in line with the suggestion of Kerr and Jermier (1978) that interdependence between team members can lead to close guidance and feedback and replace the effect of leadership behaviors. If task interdependence provides the conditions for the emergence of team meaningfulness, it works in a similar direction to that of empowering efforts of leaders, because both factors contribute to team meaningfulness. In such a situation, it is likely that task interdependence will account for some portion of team meaningfulness, attenuating the contribution of empowering leadership behaviors.

In sum, we suggest that the contribution of empowering leadership to team meaningfulness is contingent on the task interdependence level. Low task interdependence provides fewer opportunities for team members to interact and understand the team’s task significance and identity and does not increase their sense of team belongingness. In such cases, the empowering team leader serves as the primary facilitator of team meaningfulness. Under high level of task interdependence, however, team members’ interactions offer more opportunities to understand the team’s task significance and task identity, and is also likely to strengthen team identity. Under these conditions, both empowering leadership and task interdependence act in the same direction concerning team meaningfulness. Therefore, high task interdependence is likely to act as a substitute for leadership behaviors and attenuate the contribution of empowering leadership to team meaningfulness and performance.

*Hypothesis 2: Task interdependence attenuates the positive relationship between empowering leadership and team meaningfulness, such that the higher the task interdependence, the weaker the relationship.*

*Hypothesis 3: Task interdependence attenuates the indirect effect between empowering leadership and team performance through team meaningfulness, such that the higher the task interdependence, the weaker the indirect effect.*

## Materials and Methods

### Sample and Procedure

Data were collected from three technology organizations in Israel. Employees (both leaders and members) who agreed to participate voluntarily filled out a web-based questionnaire delivered by e-mail. Team members evaluated empowering behaviors of leaders and team meaningfulness, whereas leaders reported task interdependence and team performance. All responses were confidential.

The initial sample consisted of 391 participants (including both leaders and team members) from 81 R&D and technology implementation teams. Only teams that met the following criteria were included in the final sample: (a) the response rate of intrateam members was at least 50%; (b) at least two team members responded; (c) the team leader responded; and (d) the minimum tenure of participants on the team was three months.

Forty-seven teams met all the specified criteria and were included in the final sample. The R&D and technology implementation teams consisted of 263 participants (47 leaders and 216 members). The mean team size was 8.06 members (*SD* = 5.78, median = 6.00). Response rates of members ranged from 50% to 100%, with a mean of 74% (*SD* = 17.72) and a median of 71%.

Among the leaders, 89% were men, the mean age was 43.24 years (*SD* = 9.60), the mean organizational tenure was 12.87 years (*SD* = 9.94), and the mean team leadership tenure was 3.44 years (*SD* = 2.79). Among the members, 75% were men, the mean age was 38.07 years (*SD* = 9.48), the mean organizational tenure was 8.87 years (*SD* = 9.06), and the mean team membership tenure was 3.51 years (*SD* = 4.68).

### Measures

All the responses were reported on a 7-point Likert-type scale ranging from “strongly disagree” (1) to “strongly agree” (7).

*Empowering Leadership Behaviors* were assessed using empowering leadership scale (Ahearne et al., 2005; Zhang and Bartol, 2010). This scale has four multi-item subscales (with three items each) that focus on: (a) enhancing the meaningfulness of work (α = 0.92; example item: “My manager helps me understand how my objectives and goals relate to that of the company”); (b) fostering participation in decision making (α = 0.89; example item: “My manager makes many decisions together with me”); (c) expressing confidence in high performance (α = 0.85; example item: “My manager believes that I can handle demanding tasks”); and (d) providing autonomy from bureaucratic constraints (α = 0.80; example item: “My manager allows me to do my job my way”).

Previous studies (Zhang and Bartol, 2010) indicated that while these dimensions are distinct, they also collectively reflect the overall construct. Since our data are dependent (individuals are parts of teams), we followed Brown’s (2015) recommendation of conducting a confirmatory factor analysis (CFA). Using Mplus 8.4, we conducted a CFA with the TYPE = COMPLEX option of the ANALYSIS command. This option executes a standard, non-structured analysis in which the model goodness of fit measures and standard errors of the parameter estimates were adjusted for dependency in the data (Brown, 2015). The fit indices for the four first-order factors (the four subscales) and the second-order factor fell within an acceptable range [χ^2^ (50) = 109.56, *p* < 0.01; comparative fit index (CFI) = 0.94; Tucker–Lewis index (TLI) = 0.92; standardized root mean square residual (SRMR) = 0.053], which allowed us to use the total measure of empowering leadership (α = 0.94).

*Task interdependence* was measured using Barrick et al.’s (2007) four-item task interdependence scale, based on Campion et al. (1993) (example item: “Within the team I lead… team members cannot accomplish their work without information or materials from other members of their team”). The Cronbach’s alpha reliability was calculated to be 0.89.

*Team meaningfulness* was measured using the three-item subscale of team meaningfulness taken from Kirkman et al. (2004a) team empowerment measure (example item: “My team believes that its projects are significant”). The Cronbach’s alpha reliability of this scale was calculated to be 0.93.

*Team performance* was measured using the five-item team performance scale developed by Kirkman and Rosen (1999) (example item: “My team completes its tasks on time”). The Cronbach’s alpha reliability was calculated to be 0.76.

#### Control Variables

We controlled for possible differences between the three organizations, which were all technology companies based in Israel, and for team size. In addition, since previous studies indicated that gender and education level of leaders could impact team outcomes (e.g., Rowold, 2011), these variables were also controlled. Finally, we controlled for team demographics, specifically age and gender (Lee et al., 2018b), by using age diversity in the team (as expressed by age standard deviation of team members) and the proportion of women in the team (Chattopadhyay et al., 2004).

#### Aggregation to the Team Level

Empowering leadership behaviors and team meaningfulness were measured using reports of followers. To analyze the research model at the team level, we aggregated the mean scores of the team for these two variables. Following Bliese ’s (2000) recommendation, we used both the within-group coefficient of agreement [Rwg(j)] and intraclass correlations (ICCs) to justify the aggregation of the data at the team level. As a preliminary step, ANOVA was used to contrast the within-group variance from the between-group variance.

The results revealed sufficient levels of Rwg(j) indicators for empowering leadership (mean = 0.88; SD = 0.21; median = 0.94) and team meaningfulness (mean = 0.83; SD = 0.27; median = 0.94). Intraclass correlations for empowering leadership were [ICC(1) = 0.10, *F*(46,169) = 1.47, *p* < 0.05; ICC(2) = 0.32] and for team meaningfulness were [ICC(1) = 0.17, *F*(46,169) = 1.92, *p* < 0.01, ICC(2) = 0.48]. The Rwg(j) values were above the critical cutoff value of 0.70 (James et al., 1984). The ICC(1) values exceeded 0.05 (Bliese, 2000) and were statistically different from zero (Chen et al., 2004). These results suggest that it is appropriate to aggregate individual responses at the team level. Lastly, to ensure that empowering leadership and team meaningfulness were independent factors, we applied CFA (with the TYPE = COMPLEX option) on a two-factor model (considering the second-order factor construct of empowering leadership). All standardized factor loadings of the latent variables on their indicators were significant (*p* < 0.01), ranging from 0.61 to 0.96. Furthermore, fit indices provided evidence of an acceptable fit (χ^2^(85) = 168.95, *p* < 0.01; CFI = 0.95; TLI = 0.94; SRMR = 0.059). A comparison of the two-factor model with the one-factor model (χ^2^(90) = 846.80, *p* < 0.01; CFI = 0.52; TLI = 0.44; SRMR = 0.120) with respect to their chi score difference revealed a better fit for the two-factor model (Δχ^2^(5), *p* < 0.01).

## Results

### Descriptive Statistics

Table 1 presents the means and standard deviations for all variables, and the correlation matrix of all these variables at the team level.

**TABLE 1:** Descriptive statistics and intercorrelations for study variables.

Variables	*M*	*SD*	1	2	3	4	5	6	7	8	9
1. Company	2.04	0.69	–								
2. Team size	8.06	5.78	−0.34*	–							
3. Leaders’ gender	1.11	0.31	−0.02	0.03	–						
4. Leaders’ education	3.15	0.83	−0.57**	0.02	0.27	–					
5. Members’ age diversity	5.41	3.53	−0.09	0.13	−0.22	−0.21	–				
6. Proportion of women	0.23	0.28	−0.08	−0.09	0.34*	0.15	−0.06	–			
7. Empowering leadership	5.66	0.62	−0.18	−0.29*	−0.21	0.17	−0.07	0.01	–		
8. Task interdependence	4.65	1.50	−0.23	0.09	−0.10	0.09	0.22	−0.15	0.11	–	
9. Team meaningfulness	5.76	0.73	−0.14	−0.33*	−0.16	0.21	0.11	−0.17	0.43**	0.37*	–
10. Team performance	5.79	0.72	−0.15	0.02	−0.13	−0.07	0.16	0.03	0.01	0.20	0.41**

N = 47, *p < 0.05, **p < 0.01. Gender: 1, Male; 2, Female. Education: 1, High school or equivalent; 2, Diploma or equivalent; 3, B.A. or equivalent; 4, M.A. or equivalent; 5, Ph.D. or equivalent.

### Hypothesis Testing

Data were analyzed at the team level using a hierarchical linear regression model and PROCESS (Hayes, 2018). Linear regression results showed a positive relationship between empowering leadership and team meaningfulness (β = 0.29, *p* < 0.05; see Table 2, Model 2) and between team meaningfulness and team performance (*F* = 2.18, *p* < 0.05; β = 0.48, *p* < 0.05). Using a 5,000-replication bootstrap sample with 95% bias-corrected CI (PROCESS, Model 4, Hayes, 2018), and controlling for company, team size, gender of leaders, education of leaders, age diversity of members, and proportion of women, we found support for the indirect effect predicted in Hypothesis 1. Empowering leadership was found to have a positive indirect relationship with team performance through team meaningfulness (*B* = 0.22, *SE* = 0.14, 95% CI [0.01, 0.54]).

**TABLE 2:** Hierarchical linear regression models for team meaningfulness.

	Model 1	Model 2	Model 3
Company	–0.15	0.01	0.14
Team size	−0.41**	−0.28^†^	−0.21
Leaders’ gender	–0.11	−0.03	0.02
Leaders’ education	0.22	0.21	0.31^†^
Members’ age diversity	0.17	0.14	0.13
Proportion of women	–0.20	−0.16	−0.10
Empowering leadership		0.29*	0.32*
Task interdependence		0.26^†^	0.25^†^
Empowering leadership × Task interdependence			−0.30*
*F* value	2.49*	3.16**	3.56**
*R* ^2^	0.16	0.27	0.33
Δ*R*^2^		0.11*	0.06*

N = 47, ^†^p < 0.1, *p < 0.05, **p < 0.01. Standardized coefficients are reported.

Hypothesis 2 predicted that task interdependence will moderate the positive relationship between empowering leadership and team meaningfulness, such that the higher the task interdependence, the weaker the relationship. To test this hypothesis, we used a hierarchical regression method. Both empowering leadership behaviors and task interdependence were centered to reduce multicollinearity between them (Preacher and Rucker, 2003). As presented in Table 2, Model 3, task interdependence interacted with empowering leadership on team meaningfulness (β = −0.30, *p* < 0.05).

**FIGURE 2:**
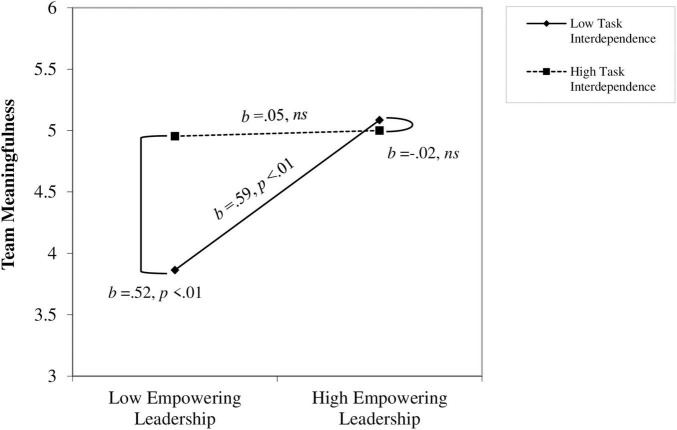
Interaction between empowering leadership behaviors and task interdependence and its effect on team meaningfulness.

To probe the nature of the interaction, we conducted a simple slope analysis (Aiken and West, 1991). This analysis revealed that when task interdependence was low (-1*SD*), empowering leadership behaviors were positively related to team meaningfulness (*b* = 0.59, *t* = 2.96, *p* < 0.01); however, when task interdependence was high (+1*SD*), the relationship between empowering leadership behaviors and team meaningfulness was not significant (*b* = 0.05, *t* = 0.26, *ns*; see Figure 2). These results support Hypothesis 2. An additional finding, as seen in Figure 2, is a significant positive relationship between task interdependence and team meaningfulness at low levels of empowering leadership behaviors (-1*SD*; *b* = 0.52, *t* = 2.92, *p* < 0.01), however, this relationship is not significant at high levels of empowering leadership behaviors (+1*SD*; *b* = −0.02, *t* = −0.10, *ns*).

To test Hypothesis 3, which predicted an indirect relationship between empowering leadership and team performance through team meaningfulness at two levels of task interdependence (1 *SD* below and 1 *SD* above the mean), we used a moderated mediation model with PROCESS (Model 7, 5,000 bootstrap resamples; Hayes, 2018) while controlling for company, team size, gender of leaders, education of leaders, age diversity of members, and proportion of women. The results revealed a significant indirect effect when task interdependence was low (*B* = 0.40, *SE* = 0.21, 95% CI [0.02, 0.84]), but not when task interdependence was high (*B* = 0.05, *SE* = 0.12, 95% CI [-0.17, 0.36]). These results support Hypothesis 3.

## Discussion

In light of the changing nature of work design to be more socially embedded and interdependent than ever before, and the growth of teamwork in organizations (Grant and Parker, 2009; Parker et al., 2013; Mathieu et al., 2017), the current study draws attention to task interdependence as a focal team state that moderates the relationship between leadership behaviors and team meaningfulness and performance. Thus far, most research on meaningfulness has focused on the individual level, and the limited literature on cultivating team meaningfulness has explored the topic only as part of empowering leadership (Kirkman and Rosen, 1999; Kirkman et al., 2004a). We address this gap by exploring how team task interdependence may affect the relationship between empowering leadership behaviors and team meaningfulness. Our results show that the direct relationship between empowering leadership and team meaningfulness, and an indirect relationship with team performance, only exist when task interdependence is low. This supports the idea that high task interdependence acts as a substitute for empowering leadership behaviors. Team task interdependence attenuates the effect of leaders’ efforts because it works in the same direction concerning the outcome of team meaningfulness and performance. Additional support for this claim can be found in the findings of a positive relationship between task interdependence and team meaningfulness when empowering leadership is low.

These findings offer theoretical contributions to the literature on leadership, meaningfulness in teams, and relational work design. We further contribute to empowering leadership literature by integrating ideas from relational work design (Grant and Parker, 2009) with the substitute for leadership framework to explain when social characteristics of work design can be a substitute for empowering leadership behaviors. The finding that empowering leadership behaviors can enhance team meaningfulness, which, in turn, leads to higher team performance, is in line with previous findings on the more general concept of team empowerment (Kirkman and Rosen, 1999; Kirkman et al., 2004a; Lee et al., 2018a). However, we showed that high levels of task interdependence can also be a substitute for the contributions of empowering leadership behaviors toward team meaningfulness. While doing so, we specified the boundary conditions and a possible moderator for empowering leadership effectiveness. Moreover, we demonstrated how the team task interdependence, which enhances interactions of team members, can be a substitute for behaviors of leaders that foster emergent team states (e.g., meaningfulness) and outcomes (e.g., performance). Kozlowski and Bell (2013) argued that any research that fails to consider task interdependence with regard to the team phenomenon in question “has little relevance to building knowledge in the work groups and teams literature. It is a feature that should be explicitly addressed—either as a boundary condition or a moderator—in all research on work groups and teams” (p. 70). Wageman (2001) found that although research emphasizes the coaching roles of leaders, interdependence can be a more critical part of leaders’ team design choices for team performance. Hence, our findings suggest that when organizations have the chance to design their team tasks in a highly interdependent fashion, enhancing interactions of members in processes such as planning and coordinating taskwork (Courtright et al., 2015), it may reduce the dependency on empowering behavior of leaders to foster team meaningfulness. These findings raise an interesting question regarding the roles of leaders when team interdependence is high. Research suggests that, alongside the positive effect of empowerment on performance, a burdening process due to increased autonomy and task complexity can increase job-induced tension of followers, and diminish the positive influence of empowering leadership on their performance (Langfred and Moye, 2004; Cheong et al., 2016). Moreover, research also shows that the effect of team autonomy on performance is contingent on the level of task interdependence (Langfred, 2005). Thus, when team task interdependence is high, the roles of leaders may include easing the tension of members by supplying sufficient and appropriate resources to help them complete their tasks while managing team autonomy to fit the level of task interdependence to enhance performance. Future studies can explore the roles of leaders in such situations and other possible roles that can contribute to team meaningfulness and performance when team task interdependence is high.

The nature of the teams participating in our study could partly explain the strong substitute for leadership effect that we found when team interdependence was high. In our research, the teams were ongoing, professional, and consisted of experienced members. These team members worked together for extended periods (at least 3 months) on tasks involving long work cycles, and were expected to work together on future tasks. Compared with temporary team members, members of ongoing teams tend to be more focused on interpersonal relationships and social interactions related to interdependence (De Jong and Elfring, 2010). Moreover, the team members in our study were all professionals who differed from non-professionals in terms of their intrinsic task satisfaction and the motivation factors that serve as strong substitutes for leadership (Howell and Dorfman, 1986).

This study also contributes to the work meaningfulness literature. Previous research has focused on the behaviors of leaders as facilitators of team meaningfulness. This study, however, uses task interdependence as a feature of relational work design that enhances the interaction of team members and demonstrates that it enables the emergence of team meaningfulness and can substitute empowering leadership behaviors. Our results are in line with those of previous studies, which showed that high interdependence is a driver of meaningful taskwork-related interactions and processes that contribute to task-related emergent states (Courtright et al., 2015).

Finally, this study contributes to the relational work design literature. The initial empirical efforts within this literature found that interactions of workers with their service beneficiaries outside the organization enhance the perceived significance of their work and performance (e.g., Grant et al., 2007; Grant, 2008b). Parker et al. (2013) extended this with a study that showed that internal structural interdependencies among employees in a department provided greater support, and enhanced job and role outcomes for them. The current study further extends this literature to include the work structure of teams, showing that greater interdependencies between team members can substitute the contribution of empowering leaders to team meaningfulness and performance.

### Managerial Implications

Our study offers practical implications for managers seeking to enhance team meaningfulness and performance. It suggests that their leadership choices should seek to balance their coaching efforts with the task interdependence design of their teams (Wageman, 2001). While facing managerial decisions related to team task design, leaders should be aware that designing their team tasks in a highly interdependent manner can contribute to team meaningfulness and performance. In the long run, this can make their teams more autonomous and less dependent on empowering behaviors of leaders. By designing teams and tasks to be more interdependent, leaders may build teams with more resource interdependence so that team members can be encouraged to depend more on one another for access to critical resources. Alternatively, they may design the process to be highly interdependent to enhance interconnectedness by creating workflows that require coordinated action (Courtright et al., 2015). For example, a manager could design an iterative or reciprocal task workflow instead of assigning team subtasks that must be completed by team members individually. However, if the team task does not require high levels of interdependence (e.g., pooled, mindless, or reactive execution of work), or when the team’s emergent level of interdependence is low (Wageman, 2001), empowering behaviors of leaders are essential for fostering team meaningfulness and performance.

### Limitations and Future Research

This study is not without limitations. First, our sample was based on technology organizations. Testing our model in other team-based environments spanning different industries and sectors, such as low-tech and non-profit organizations, can contribute to the generalization of the findings. Second, although we examined two different sources in our research model (team members and leaders), and all of the teams were ongoing, this study was cross-sectional in design. Future studies may take advantage of a time-lagged or longitudinal design to examine the processes in our model. Third, our sample exhibited a high degree of homogeneity in terms of gender (89% of leaders were men, as were 75% of members). This level of male dominance can be found in many technological organizations, and we, therefore, controlled for the gender proportions of team members. Future studies could test the model in organizations with more gender-balanced teams.

Our findings suggest several directions for future research. First, we explored the relationship between task interdependence and team meaningfulness in ongoing professional teams, measuring the perception of teamwork as significant, worthwhile, and meaningful (Kirkman et al., 2004a). Future research should explore other types of teams (e.g., project or *ad hoc* teams) to better understand how task interdependence contributes to their team meaningfulness. Second, task interdependence may substitute empowering effect of leadership behaviors on team meaningfulness through several plausible paths, namely, task significance, task identity, and a sense of team identification. We encourage future studies to explore these mediating mechanisms. Finally, apart from task interdependence, outcome interdependence, along with other structural and compositional features of team relational design, such as team diversity (Grant and Parker, 2009; Mathieu et al., 2017), offers a path for future research on antecedents or moderators that may enhance or attenuate team meaningfulness. One particular structural feature that may impact meaningfulness, which has increased rapidly since the COVID-19 pandemic crisis, is team virtuality. Thus, future research should provide insights into ways of enhancing team meaningfulness in virtual teams.

## Conclusion

By relying on ideas from the relational work design perspective and substitute for leadership, this study explores team task interdependence as a substitute for empowering impact of leaders on team meaningfulness and performance. We demonstrated that empowering leadership contributes directly to team meaningfulness and indirectly to team performance at low but not high task interdependence levels, thus indicating that task interdependence can substitute for empowering leadership.

## Data Availability Statement

The raw data supporting the conclusions of this article will be made available by the authors, without undue reservation.

## Ethics Statement

The studies involving human participants were reviewed and approved by the Human Subjects Research Committee of Ben-Gurion University of the Negev. The patients/participants provided their written informed consent to participate in this study.

## Author Contributions

AL contributed to the theoretical development and empirical aspects of this study, data analysis, and preparation of this manuscript. RH contributed to the theoretical development of this study as well as to the preparation of the manuscript. TI contributed to the theoretical development, reviewed this manuscript critically, and gave important intellectual input. SH contributed to the initial theory development, research design, and data collection. All authors contributed to the article and approved the submitted version.

## Conflict of Interest

The authors declare that the research was conducted in the absence of any commercial or financial relationships that could be construed as a potential conflict of interest.

## Publisher’s Note

All claims expressed in this article are solely those of the authors and do not necessarily represent those of their affiliated organizations, or those of the publisher, the editors and the reviewers. Any product that may be evaluated in this article, or claim that may be made by its manufacturer, is not guaranteed or endorsed by the publisher.
